# Selenium nanoparticles produce a beneficial effect in psoriasis by reducing epidermal hyperproliferation and inflammation

**DOI:** 10.1186/s12951-021-00842-3

**Published:** 2021-04-13

**Authors:** Vinod Gangadevi, Sowjanya Thatikonda, Venkatesh Pooladanda, Geetanjali Devabattula, Chandraiah Godugu

**Affiliations:** Department of Regulatory Toxicology, National Institute of Pharmaceutical Education and Research (NIPER), Balanagar, Hyderabad, Telangana 500037 India

**Keywords:** Psoriasis, Selenium nanoparticles, Reactive oxygen species, Inflammation, Proliferation, Imiquimod

## Abstract

**Background:**

Psoriasis is a chronic autoimmune skin disease characterized by hyperproliferation of keratinocytes. Wide treatment options used to treat psoriasis is associated with various adverse effects. To overcome this nanoformulation is prepared. Selenium is an essential trace element and plays major role in oxidation reduction system. Toxicity and stability limits the applications of selenium. Toxicity can be reduced and stabilized upon preparation into nanoparticles.

**Results:**

Selenium nanoparticles (SeNPs) exhibit potent apoptosis through the generation of reactive oxygen species (ROS) with cell cycle arrest. SeNPs topical gel application produced significant attenuation of psoriatic severity with the abrogation of acanthosis and splenomegaly. SeNPs reduced the phosphorylation and expressions of MAPKs, STAT3, GSK-3β, Akt along with PCNA, Ki67, and cyclin-D1.

**Conclusion:**

SeNPs inhibit various inflammation and proliferation mediated pathways and could be an ideal candidate for psoriasis therapy.

**Materials and methods:**

SeNPs were characterized and various techniques were used to determine apoptosis and other molecular mechanisms. In vivo studies were performed by inducing psoriasis with imiquimod (IMQ). SeNPs were administered via topical route.

**Graphic Abstract:**

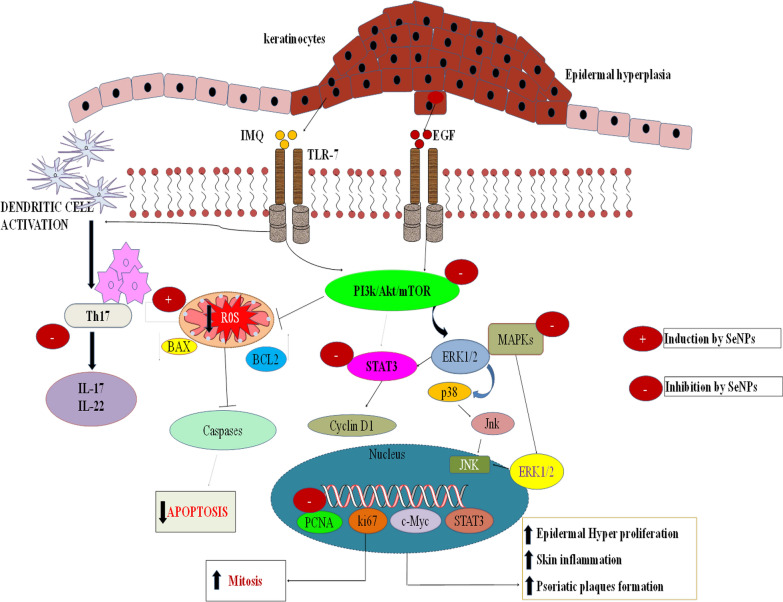

## Introduction

Psoriasis is an autoimmune chronic skin disease characterized by hyperproliferation of keratinocytes [1]. The amplification of dendritic cells and T-cells from the immune system releases various proinflammatory cytokines and chemokines with simultaneous activation of growth factors together involve in the pathogenesis of psoriasis [2]. The main histological and characteristic features of psoriasis are acanthosis, prominent dilated blood vessels in the dermis, and leukocyte infiltration [3]. Psoriatic keratinocytes possess an enhanced ability to endure apoptosis with a dramatic increase in cell cycle progression, which results in the elongation of rete ridges that protrudes into the dermis [4, 5]. Epidemiological evidence suggests that psoriasis is affecting 2–3% population which is associated with pain and pruritus [6]. Moreover, psoriasis patients suffer from impaired quality of life with emotional distress and the burden of comorbidities such as hypertension [7], diabetes mellitus [8] and obesity [9] with the increase in the psoriasis severity.

The activation of T helper (Th) cells including Th1, Th17, and Th22 cells trigger the inflammatory process of psoriasis and upregulate an array of proinflammatory cytokines [10], among, Th17 cells are the primary source of IL-22, which principally targets keratinocytes and orchestrates its effect through the activation of mitogen activated protein kinases (MAPKs) and signal transducer and activator of transcription 3 (STAT3) [11]. In keratinocytes, the growth factors which include epidermal growth factor (EGF), vascular endothelial growth factor (VEGF), and keratinocyte growth factor (KGF) majorly contribute to reinforcing the epidermal hyperplasia, angiogenesis and cell differentiation [12].

Topical corticosteroids remain the mainstay in psoriasis therapy with different potencies, but their long term usage leads to various adverse effects [13]. Although psoralen with UV-A (PUVA) is the effective psoriasis treatment, its use is diminishing because of its interconnection with malignancies. Methotrexate, tacrolimus, acitretin, and cyclosporine-A are the commonly used systemic or topical agents with high efficacy but prolonged usage is associated with systemic toxicity and other inherent side effects [14]. Biologicals such as adalimumab, infliximab, secukinumab, and ustekinumab are successful therapeutic options for psoriasis with good efficacy, for moderate to severe psoriasis [15]. Still, there is a substantial unmet clinical need to develop safer therapeutic approaches for long term usage.

Nanomedicine has evolved as a distinct method of treatment in therapeutics by overcoming the problems associated with conventional drug forms [16, 17]. Various other nanoparticle systems were used in treating psoriasis such as methotrexate containing gold nanoparticles [18] and silver nanopaticles [19]. The nanoparticles novel drug delivery systems offer enhanced therapeutic activity with fewer side effects in treating psoriasis [20]. Metal nanoparticles like Se, Au, Ag, Zn, Ce, and Fe are widely used in various ailments, among which SeNPs are extensively used because of their captivating applications. [21]. Se exists as Se^2+^, Se^4+^, Se^6+^, Se^2−^ oxidation forms and its toxicity can be reduced upon the preparation into nanoparticle formulations [22]. SeNPs can be synthesized by various biological methods (i.e. from bacteria, fungi, plants) and other synthetic methods [23, 24]. SeNPs has been explored as potent anticancer agents which increase the generation of ROS by the leakage of mitochondrial proteins and endoplasmic reticulum stress, leading to DNA damage-induced apoptosis [25]. SeNPs in combination has been found to overcome the systemic resistance of various chemotherapeutics such as 5- fluorouracil, irinotecan, and adriamycin with effective synergistic activity [26, 27]. On the other side, SeNPs are found to attenuate certain drug-induced toxicities like thyrotoxicity, nephrotoxicity, and genotoxicity [28]. Also, SeNPs are effective in treating the inflammation-driven disorders such as arthritis, liver fibrosis, as well as nephropathy, and the main mechanism behind the anti-inflammatory activity is through the reduction of inflammatory cytokines levels such as IFN-ϒ, IL-1β, IL-12, IL-6, and IL-2 [29].

The current study aims to investigate the potential role of SeNPs in alleviating epidermal acanthosis and inflammation in psoriasis. SeNPs may shed light as a promising therapeutic treatment option on the proliferation and inflammation-driven psoriasis.

## Materials and methods

### Chemicals and reagents

Formaldehyde, Glycine, Potassium chloride, Tris–HCl, Dimethyl sulphoxide (DMSO), Bicinchonic acid (BCA), Sodium dodecyl sulphate (SDS), Propidium iodide (PI), reduced glutathione (rGSH), 2′, 7′-dichlorodihydrofluorecein diacetate (DCFDA), 3-(4,5-dimethylthiazol-2-yl)-2,5-diphenyl tetrazolium bromide (MTT), 5,5-dithio-bis(2-nitrobenzoic acid) (DTNB), Sodium hydroxide were procured from Sigma-Aldrich, USA. Enzyme-linked immunosorbent assay (ELISA) kits for TGF-β, IL-22, IL-1β, IL-17A, and IL-6 were procured from Thermo scientific, USA. Enhanced chemiluminescence (ECL) reagent and nitrocellulose membrane were purchased from Biorad, USA. Fetal bovine serum (FBS), Roswell park memorial institute-1640 (RPMI-1640) medium, Antibiotic–Antimycotic and Trypsin–EDTA were purchased from GE Health care, USA. The primary antibodies such as anti-p-MAPK p44/42, anti-MAPK p44/42, anti-p-SAPK/JNK, anti-SAPK/JNK, anti-p-MAPK p38, anti-MAPK p38, anti-p-GSK-3β, anti-GSK-3β, anti-p-Akt, anti-Akt, and anti-Cyclin D1 were purchased from Cell Signaling Technologies, USA. Anti-β-Actin and anti-Ki67, anti-mTOR, anti-p-STAT3, anti-PCNA as well as secondary antibodies such as anti-Rabbit and anti-Mouse antibodies were procured from Santa Cruz Biotechnology, USA. The anti-STAT3 antibody was obtained from Biorad, USA. Imiquimod cream (Marketed as Imiquad cream, 5% w/w, Glenmark Pharmaceuticals Ltd., India) was purchased. All other chemicals were obtained commercially which were pure and analytical grade.

### Preparation and characterization of SeNPs

SeNPs were prepared as per the procedure described earlier [30, 31]. Briefly, 5 ml of 25 mM Sodium selenite (Na_2_SeO_3_) was mixed with 20 ml of 25 mM rGSH containing 200 mg bovine serum albumin (BSA). The pH of the obtained mixture was adjusted to 7.2. The red-colored solution obtained was dialyzed for 96 h against double distilled water to separate GSSG and free sodium selenite from SeNPs using the magnetic stirrer by changing the water every 24 h. The SeNPs suspension was taken from the dialysis bag into a 50 ml falcon tube and was subjected to centrifugation at 14,000 rpm for 10 min. The supernatant was disposed and 20 ml of double-distilled water was added to the pellet and vortex for 1 min and subjected to centrifugation at 14,000 rpm for 10 min and repeated this for 3–4 times to remove possible free sodium selenite and other reactants in nanoparticles. The size measurements were performed using the zeta analyzer (Malvern, UK). To determine the morphology and size, Transmission electron microscopy (TEM) (Hitachi, H-7500) was used. SeNPs were mixed and a small drop of SeNPs sample was taken and placed on parafilm, then the carbon-coated copper grid was placed for 5 min. Then the excess of NPs was dried, and stained with 2% uranyl acetate for negative stain contrast. After preparation of nanoparticles the UV absorption was measured at 205 nM using UV spectrophotometer by diluting water and dilution ratio was 1:10,000 and for the powder X-ray Diffraction (PXRD) patterns of samples were recorded by using x-ray diffractometer (Philips X’pert MPD System) as per the previous method described [32]. Samples were illuminated using Ni-filtered Cu-K at current of 30 mA with 40 kV voltages. Data were obtained over 2θ scan ranging from 10 to 50 with a step time of 0.012°steps.

### Topical gel formulation of SeNPs

SeNPs were formulated as a gel for topical application with adequate viscosity. 0.75% Carbapol gel was prepared by mixing accurately weighed carbapol grade 934 in distilled water with continuous stirring and the gel is neutralized using triethanolamine with pH 6.8 until a homogenous gel formation is achieved. Further, the gel is allowed to swell for 2 h. Once the adequate consistency is achieved the specified amount of SeNPs were incorporated into the gel with continuous agitation. Further 100 mg of gel which contain SeNPs 100 and 300 μg, respectively was topically applied on to the dorsal region of mice.

### Cell culture

Human keratinocytes (HaCaT) cell line was obtained as a kind gift sample from Dr. Munia Ganguly, IGIB New Delhi, India. The cells were grown in RPMI-1640 medium by supplementing with 10% FBS and 1% antibiotic–antimycotic. Further cells were incubated at 37 °C with 98% relative humidity and 5% CO_2_. Cells were subcultures when attained 80–90% confluence.

### Determination of cytotoxicity by MTT assay

The cytotoxicity induced by SeNPs was determined by MTT assay [33]. This assay detects the formation of formazan crystals by dehydrogenase enzymes of mitochondria and the cytotoxicity was determined based upon the absorbance [34]. Briefly, HaCaT cells were grown in 96 well plates and cells were treated with serially diluted SeNPs by two-fold dilutions with the concentrations ranging from 0.39 to 50 μg/ml and incubated up to 24 h. Later cells were washed with PBS for the complete removal of SeNPs. Then MTT reagent (0.5 mg/ml) was mixed in respective media and incubated for 4 h. DMSO was used to solubilize the formazan crystals and absorbance was measured at 570 nm (Spectramax M4, Molecular devices, USA).

### Microscopic evaluation of apoptosis

Acridine orange (AO) staining evaluates the morphological changes in apoptotic cells by formation of blebs in the membrane, fragmentation and condensation of the nucleus. In AO staining, AO pervade all the cells and makes the nuclei appear green. DAPI (4′, 6-diamidino-2-phenylindole) is a nuclear stain and is frequently used as nuclear segmentation tool. The changes in the nucleus can be visualized in a fluorescent microscope, which is indicative of apoptosis [35, 36]. To visualize these changes, HaCaT cells were plated in 12-well plates and the following next day, cells were incubated with SeNPs at the concentrations of 0.5, 1 and 2.5 µg/ml. After 1 h, EGF (50 ng/ml) had been stimulated and further incubated for 24 h, then stained with AO and DAPI. Then the images were captured at ×200 by fluorescent microscope (Nikon Eclipse TiS, Japan).

### Cell cycle analysis

PI staining was performed to evaluate the induction of cell cycle arrest in epidermal keratinocytes and then analyzed by flow cytometry [37]. In this method, HaCaT cells were seeded in 12 well plates and treated with SeNPs for 1 h followed by EGF induction and incubated further for 24 h. After trypsinization, the cells were fixed in 70% ethanol and stored at – 20 °C. Later the ethanol fixed cells were washed with PBS and incubated for 15 min in staining buffer which contains triton-X-100, PI, and RNAse enzyme) and then cells were vortexed and analyzed by flow cytometry (BD C6 Accuri flow cytometry, USA).

### JC-1 staining

The modulation in the redox potential of the mitochondrial membrane can be determined by JC-1 staining [38]. The shift in the emission of fluorescence will be observed from red to green, which depends on mitochondrial membrane potential (ΔΨm). Green fluorescence intensity develops due to “J monomers” which occurs at depolarized ΔΨm, while red fluorescence intensity develops due to “J aggregates”, which are formed at high membrane potentials [39]. To test this effect, in this assay, HaCaT cells were plated at a cell density 4 × 10^4^ in 12 well plates, and treated with SeNPs and incubated for 24 h in the presence of EGF stimulation. Later, these cells were stained with JC-1 dye (1 µM for 30 min) and it was quantitatively analyzed by flow cytometry.

### Alexa Fluor 488 Annexin V/Dead cell apoptosis assay

Dead cell apoptosis assay is quick and suitable method for identification of apoptotic cells as Annexin V, being phospholipid binding protein has more affinity towards phosphatidyl serine, which moves from inner plasma membrane leaflet to outer leaflet in apoptotic cells and thereby these apoptotic cells can be quantitatively estimated [40] For this assay, Alexa Fluor® 488 Annexin V/Dead cell apoptosis kit (Thermo Fischer Scientific, USA) was used. Initially, the assay was optimized by seeding HaCaT cells at cell density 4 × 10^4^ and allowed to adhere, and further treated with SeNPs (0.5, 1 and 2.5 µg/ml) for 1 h followed by stimulation with EGF and incubated for 24 h. Then the assay was performed with aforementioned kit and followed the manufacturer protocol. Later the samples from all groups were analyzed by flow cytometry [41].

### DCFDA staining

ROS formation in the cells can be determined by DCFDA staining [30]. In this method, HaCaT cells were seeded in 6-well plates at a cell density 8 × 10^4^ and treated with SeNPs (0.5, 1 and 2.5 μg/ml) followed by EGF and incubated for 24 h. To detect ROS levels, DCFDA (10 µM) was added to the cells and incubated at 37 ℃ for 20 min in the dark. After trypsinization, cells were washed with PBS and subjected to flow cytometry. In another set of the experiment after the addition of dye, cells were visualized under the fluorescence microscope at 498/530 nm (excitation/emission) at ×200 magnification and corresponding images were captured [37].

### Experimental animals

Adult BALB/c mice (25–30 g) 6–8 weeks age were procured from Palamur Biosciences Pvt. Ltd, Mahabubnagar, India. Animals were grouped into n = 3 per cage by providing aseptic and ambient conditions, with 12 h light and dark cycle. The study protocol was approved by IAEC and all the experiments were carried out according to CPSCEA guidelines, Government of India.

### IMQ induced psoriatic plaque model

IMQ induced psoriasis-like skin inflammation is a widely accepted model that induces TLR-7/TLR-8 receptors [42]. Here, this model is used to study the effect of SeNPs on the morphological features of IMQ induced psoriasis mice. For this, mice were divided into 5 groups, consisting of 5 animals per group (n = 5). The grouping of animals were divided as Normal control group (NC), Imiquimod control group (IMQ), Standard tacrolimus group (TAC; 20 mg/kg topically), SeNPs at two doses which include low dose (SeNPL; 3 mg/kg) and high dose (SeNPH; 10 mg/kg). IMQ was applied topically at a dose of 62.5 mg/day per 5 cm^2^ and the topical treatment of SeNPs and TAC was begun from day 3 of IMQ application once daily on the shaved region of skin for 4 days and monitored daily up to 6 days. Skin fold thickness was measured by vernier calipers (Mitutoyo, Japan), on the other hand, erythema and scaling were graded independently from 0 to 4 scale (0-none; 1-slight; 2-moderate; 3-marked; 4-very marked) every alternate day on 0, 2, 4, and 6th day to obtain Psoriasis Area Severity Index (PASI) [43]. After sacrifice, the skin tissue samples were collected and stored at – 80 ℃. For histopathology analysis, the samples were fixed in 10% formalin.

### Hematoxylin and Eosin (H&E) staining

H&E staining is used to visualize the histo-pathological changes that include nuclear, cytoplasmic and epidermal changes such as epidermal hyperplasia and keratinocyte proliferation and also examine extracellular matrix features in the tissues. Hematoxylin has a deep blue-purple color which stains nucleic acids. Whereas, eosin stains cytoplasmic proteins is pink color, nonspecifically. Fixed skin tissues were dehydrated by incubating in gradient alcohol and xylene. Then 5 µm skin sections were sliced using a microtome (Leica, Germany). Further processing steps were performed according to the previous reports [24]. The images were captured at ×200 and ×400 magnification by a bright-field microscope.

### Spleen to body weight index

Animal bodyweights were recorded every day until the completion of the study and after sacrifice spleen morphology was observed and captured. To obtain spleen to body weight index, spleen weights were measured and normalized with body weights and the results were expressed in mg/g body weight.

### Immunofluorescence (IF) analysis

IF analysis involves an antibody binding to the antigens of a cell or tissue and then the bound product is visualized based on fluorescence [44]. In this method, 5 µm skin tissue sections were deparaffinized, rehydrated, washed, and treated with proteinase K for 15 min for unmasking the antigen, then 3% BSA blocking solution was used to eliminate non-specific binding portions from the sections and incubated with anti-STAT3 and anti-Ki67 (1:100 dilutions) primary antibodies overnight at 4℃. The unbound antibody from tissue sections were washed with immune wash buffer thrice and probed with Fluorescein isothiocyanate (FITC) or Rhodamine conjugated anti-rabbit or anti-mouse secondary antibodies (1:200 dilutions) at room temperature for 1 h and then sections were washed and mounted with DAPI Flour shield™ histology mounting medium (Sigma-Aldrich, USA) and the proteins expression was observed under Leica TCS SP8 Laser Scanning Spectral Confocal Microscopy (Leica, Germany).

### Estimation of cytokines by ELISA

The cytokine levels were measured by ELISA assay in skin tissue samples. Briefly, skin tissues were homogenized with Tris–HCl buffer with protease and phosphatase inhibitors and centrifuged at 12,000 rpm at 4℃ for 10 min. The supernatant from all tissues were collected and protein estimation was done by Bradford’s reagent (Biorad, USA). Polystyrene 96 well plates were coated with TGF-β, IL-17A, IL-22, IL-1β, and IL-6 along with 100 µl of antibodies diluted with coating buffer and incubated at 4℃ overnight. Later excessive and unbound antibody was removed by washing buffer and further blocked with BSA. Further 100 µl of the tissue supernatant was incubated followed by the addition of detection antibody for 1 h at room temperature. Later, 100 µl of Avidin-HRP solution was added per well and incubated for 30 min. Then 100 µl of trimethylbenzidine solution per well for 15 min and measured absorbance at 450 and 570 nm, reaction was terminated by 1 M H_3_PO_4_. The difference between absorption was calculated and protein estimated in pg/mg.

### Western blotting analysis

The western blotting was performed to study the effect of various signaling pathways involved in psoriasis. The protein was extracted from cells and skin tissues as described earlier with slight modifications [35]. Protein concentration was estimated by Bicinchonic acid (BCA) (Sigma-Aldrich, USA). Samples were loaded in SDS-PAGE (sodium dodecyl sulfate–polyacrylamide gel electrophoresis), where proteins were separated according to the molecular weight and transferred to the Nitrocellulose membrane by Trans-blot® Turbo™ Transfer starter system (Bio-Rad, USA). The membranes were placed in Ponceau stain for visualization of protein bands according to the molecular weights. Later, 3% BSA blocking solution was added to avoid nonspecific binding of antibodies, followed by primary and secondary antibodies were added, respectively and washed with TBS-T for 3 times, 5 min each and blots were detected in Chemdoc imaging system (Vilber Fusion Fx, France) by using ECL as a substrate and images were quantified by ImageJ software, NIH, USA. β-Actin was used as housekeeping protein and respective totals were used for normalization.

### Statistical analysis

Data are represented as mean ± standard deviation (SD). Graphs were plotted using Graph Pad Prism version 6 (Graph Pad Software, San Diego, CA). Statistical significance was evaluated with a one-way analysis of variance with the Bonferroni post hoc test. Values of P < 0.05 was considered as statistically significant.

## Results

### Synthesis and characterization of SeNPs

**Fig. 1:**
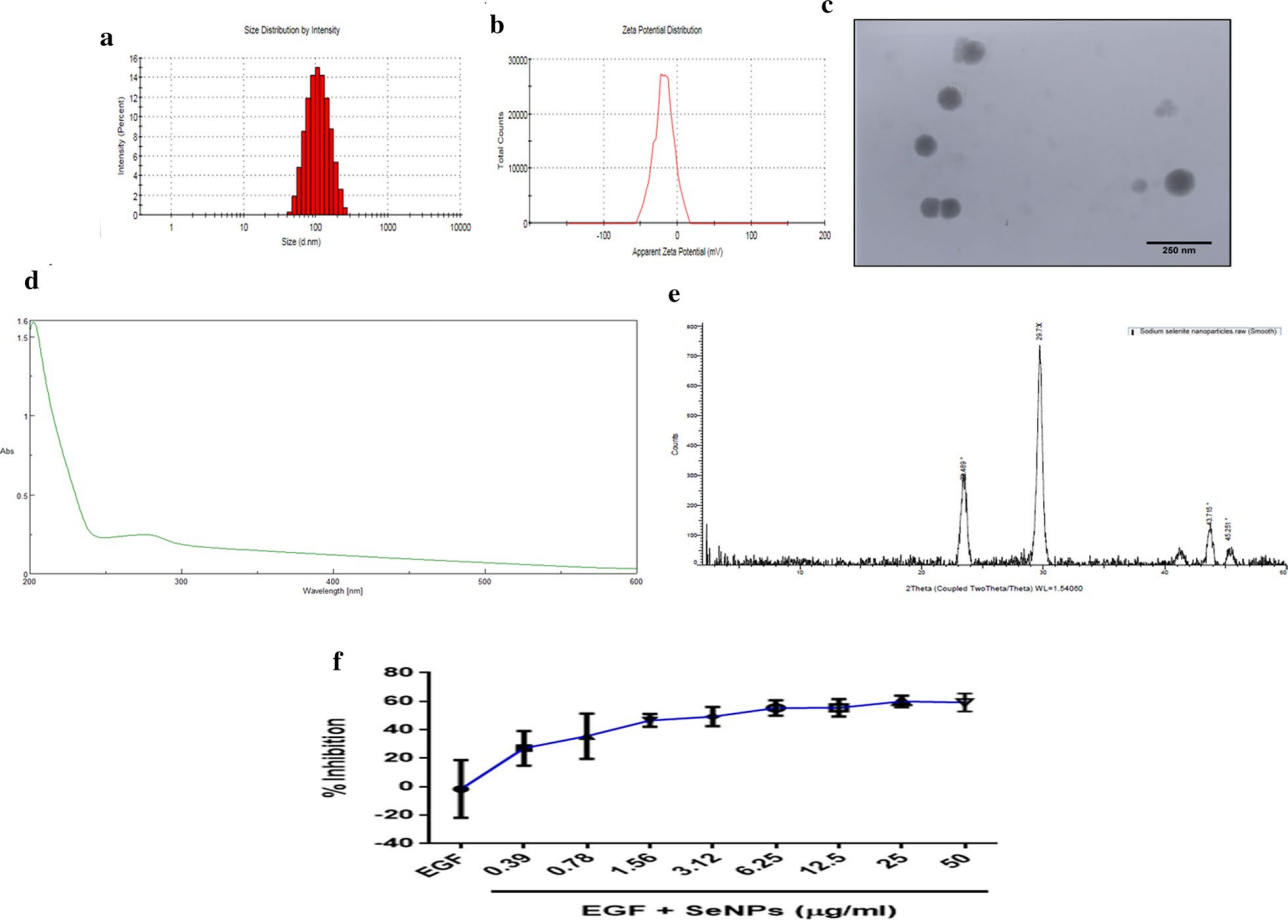
Characterization of Selenium nanoparticles (SeNPs). SeNPs were characterized and evaluated for (**a**) particle size (**b**), Zeta potential (**c**), Transmission electron microscopy. **d** The UV–Vis absorption spectrum of SeNPs. **e** XRD pattern of SeNPs. **f** Cytotoxicity of HaCaT cells was determined by MTT assay by pre-treatment of cells with SeNPs for 2 h and stimulated with EGF (50 ng/ml) then incubated for 24 h

After synthesizing of SeNPs, their size distribution was measured using Zetasizer and SeNPs were diluted (100 µl of SeNPs in 900 µl of water) to make it a uniform dispersion free of agglomerates and the average size of the SeNPs were found to be 110.73 ± 7.24 nm with a polydispersity index (PDI) of 0.146 ± 0.03 (Fig. 1a). SeNPs exhibited a negative charge with the zeta potential of – 18.50 ± 0.59 (Fig. 1b). Additionally, the size and shape of SeNPs were evaluated by TEM analysis, where SeNPs exhibited the spherical morphology (Fig. 1c). As shown in the Fig. 1d to assess the absorption of SeNPs in the visible range the UV–visible spectrum analysis was performed and the results depict that SeNPs characteristic absorption peak was obtained at 205 nm. The results from PXRD patterns depicts that the SeNPs exhibited sharp diffraction peaks as shown in the Fig. 1e which demonstrates the crystalline nature of SeNPs and these results are consistent with the previously documented data[32].

### SeNPs inhibits keratinocytes hyperproliferation by induction of apoptosis

**Fig. 2:**
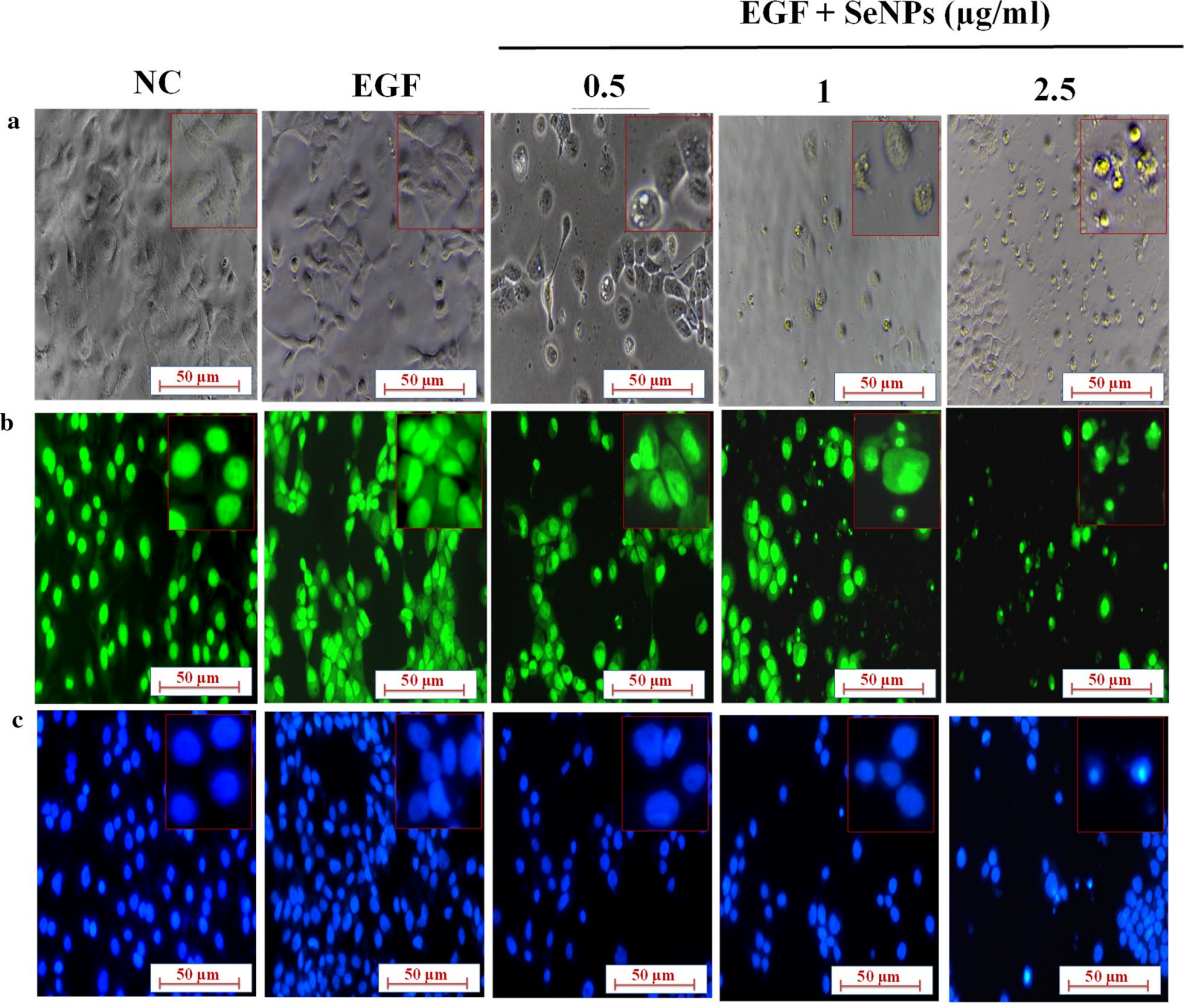
SeNPs induce apoptosis and inhibit hyperproliferation of keratinocytes. **a** SeNPs induced morphological changes visualized under phase contrast microscope. **b** Apoptotic changes induced by SeNPs by forming chromatin condensation and apoptotic bodies observed by AO/EB dual staining. **c** Nuclear and apoptotic changes were visualized by performing DAPI staining. The images were captured at × 200 magnification with fluorescent microscope

The homeostasis imbalance among cell differentiation, cell proliferation, and programmed cell death leads to keratinocyte hyperproliferation with shortened cell cycles [45, 46]. In in vitro model, the immortalized HaCaT cells were chosen, to promote hyperproliferation and evasion of apoptosis, the cells were stimulated with EGF at 50 ng/ml. To investigate the effect of SeNPs on EGF induced hyperproliferation, MTT assay was performed with SeNPs at 24 h time point at concentrations ranging from 0.39 to 50 μg/ml and further stimulated with EGF. We found a gradual concentration-dependent cytotoxicity and the IC_50_ value was found to be 3.06 ± 0.02 μg/ml (Fig. 1f). Further, the cell death mechanism induced by SeNPs was found to be through the induction of apoptosis. Morphologically phase-contrast imaging, AO and DAPI staining revealed that SeNPs treated cells significantly lost the morphology with prominent apoptotic features at 1 and 2.5 μg/ml concentration such as nucleus condensation, nuclear pyknosis, horseshoe-shaped nucleus (Fig. 2a–c).

### SeNPs induces cell cycle arrest with ROS mediated loss of ΔΨm and induction of apoptosis

**Fig. 3:**
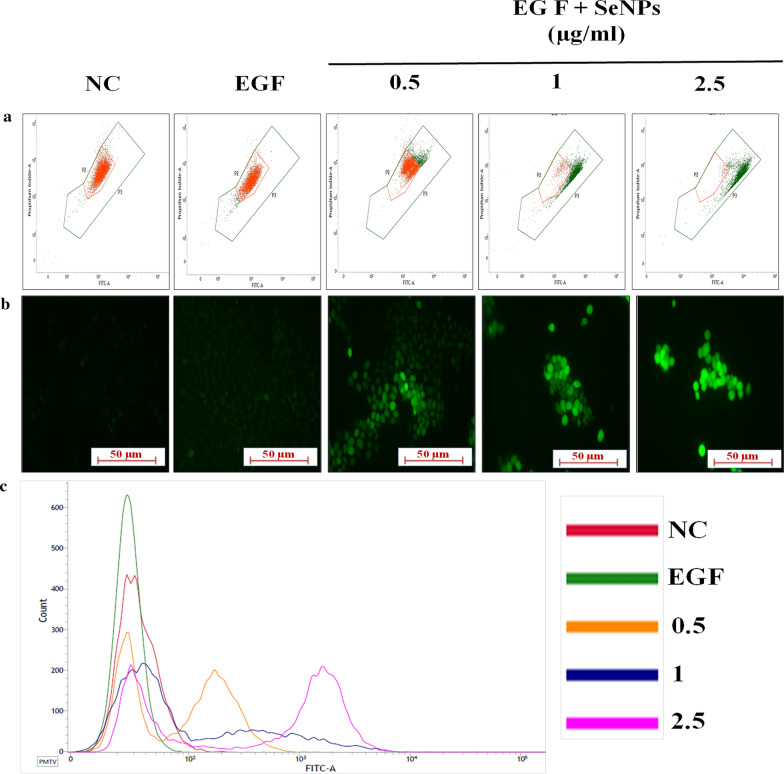
SeNPs induce the oxidative stress and mitochondrial membrane potential loss in human keratinocytes. **a** Mitochondrial membrane potential was evaluated by JC1 staining to analyze the alterations in oxidation–reduction potential of mitochondrial membrane by SeNPs. **b** The percentage J-monomers population was depicted in bar graph. Whereas, the oxidative stress induced by SeNPs was determined by DCFDA staining. **c** The fluorescent images were captured at × 200 magnification and **d** mean fluorescence intensity (MFI) was quantified and represented as bar graph. **e** Additionally, DCF fluorescent intensity was determined by flow cytometric analysis. Data represents Mean ± SD (n = 3). ^p < 0.05, ^^p < 0.01, and ^^^^p < 0.0001 are significantly different from the EGF group

**Fig. 4:**
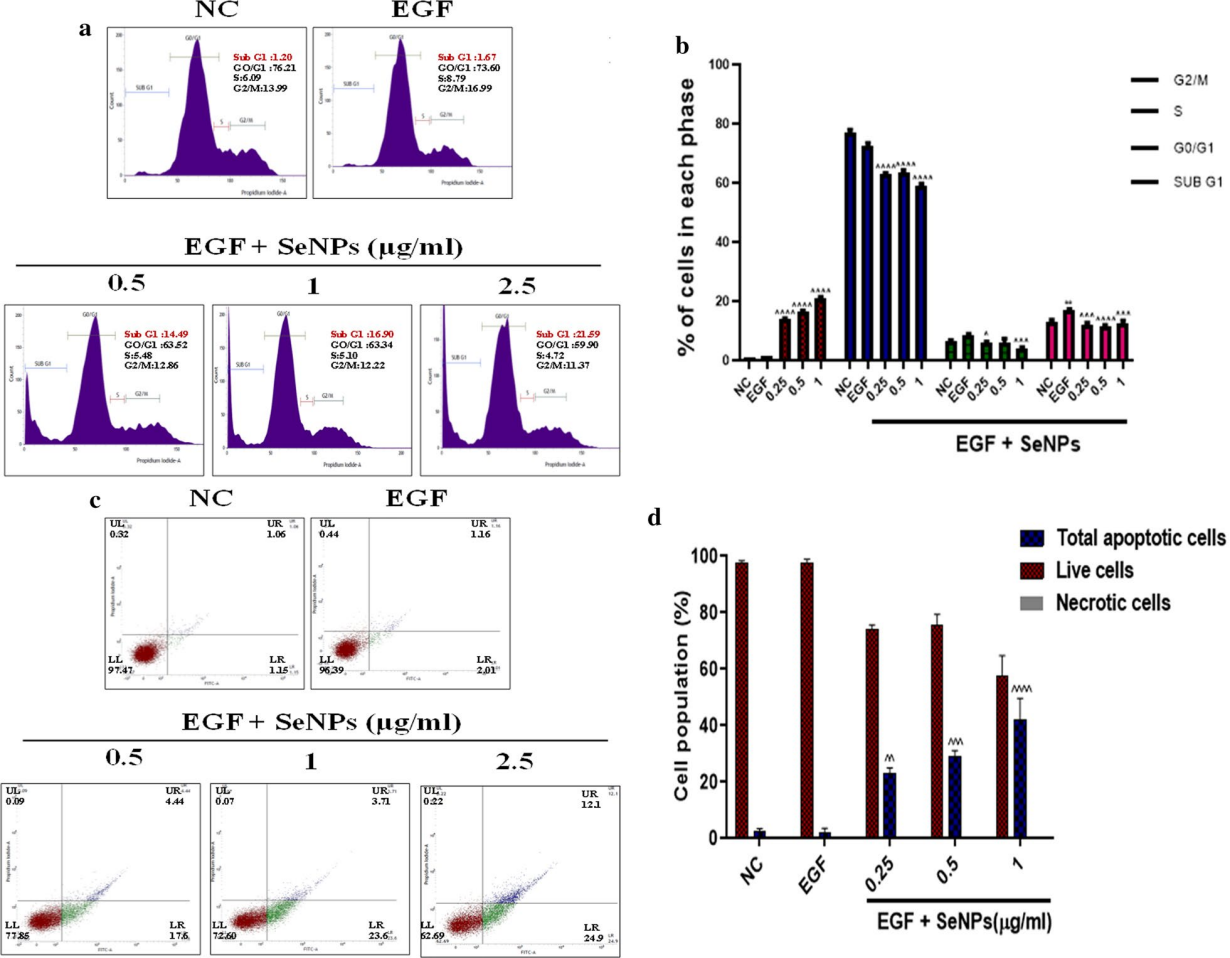
SeNPs induce the early apoptosis by arresting the cells at Sub G1 phase in keratinocytes. Induction of cell cycle arrest and apoptosis by SeNPs was observed and analyzed by flow cytometric analysis. **a** PI staining distribution in different phases of cell cycle and these peaks represent Sub G1, G0/G1, S, and G2/M phases of cell cycle in the histograms. **b** The percentage of cells was distributed in each phases were represented in bar graph. **c**, **d** Quantitative estimation of apoptosis induction was measured by Alexa Flour® 488 Annexin V staining. The percentage of cells positive for Annexin V-Alexa Flour 488 and/or PI in the quadrants was quantified. Cells in the upper left quadrant (Q1-UL; AV-/PI +): necrotic cells; lower left quadrant (Q2-LL; AV-/PI-): live cells; lower right quadrant (Q3-LR; AV + /PI-): early apoptotic cells and upper right quadrant (Q4- UR; AV + /PI +): late apoptotic cells Data represents Mean ± SD (n = 3). ^p < 0.05, ^^p < 0.01, ^^^p < 0.001 and ^^^^p < 0.0001 are significantly different from the EGF group

Hyperbaric oxygen therapy, which increases the ROS generation in skin tissues has been successfully used as a potent anti-psoriatic therapy in severe psoriasis [47]. The significant ROS generation disrupts ΔΨm and further induce the apoptotic pathway [48]. The JC-1 monomers formation as a result of the loss of ΔΨm exhibits green fluorescence, while J aggregates in control cells exhibit orange fluorescence, SeNPs treatment for 24 h resulted in a shift in the fluorescence towards green, indicating loss of ΔΨm (Fig. 3a). We next evaluated the effect of SeNPs on total cellular ROS by DCFDA staining in EGF stimulated cells. Figure 3b, c demonstrate that SeNPs treatment significantly increased ROS levels in cells, while the extent of ROS levels generated were quantified by flow cytometry, where SeNPs treated groups showed a shift of peak towards the right side. The DNA fragmentation is the strong evidence of apoptosis induction, which can be analyzed by sub G1 phase arrest. In the present study, from the flow cytometric analysis, it was found that SeNPs treatment concentration-dependently enhanced the DNA fragmentation which was detected by a sharp and discrete peak representing the subpopulation of “Sub G1” (apoptotic cells), we observed that accumulations of cells in Sub G1 peak with increased concentrations as compared to control cells. This was accompanied by a decrease in the proportion of S and G2/M phase percentages (Fig. 4a). For the quantitative assessment of apoptosis, after treatment with SeNPs for 24 h, cells were double-stained with annexin-V Alexa flour 488/PI. As illustrated in Fig. 4b, it was found that SeNPs treatment significantly increased the rate of early apoptosis (Annexin + /PI −) as well as late apoptosis (Annexin + /PI +) compared to control cells.

### SeNPs improve the phenotypic features of IMQ induced psoriatic skin lesions and attenuate splenomegaly

**Fig. 5:**
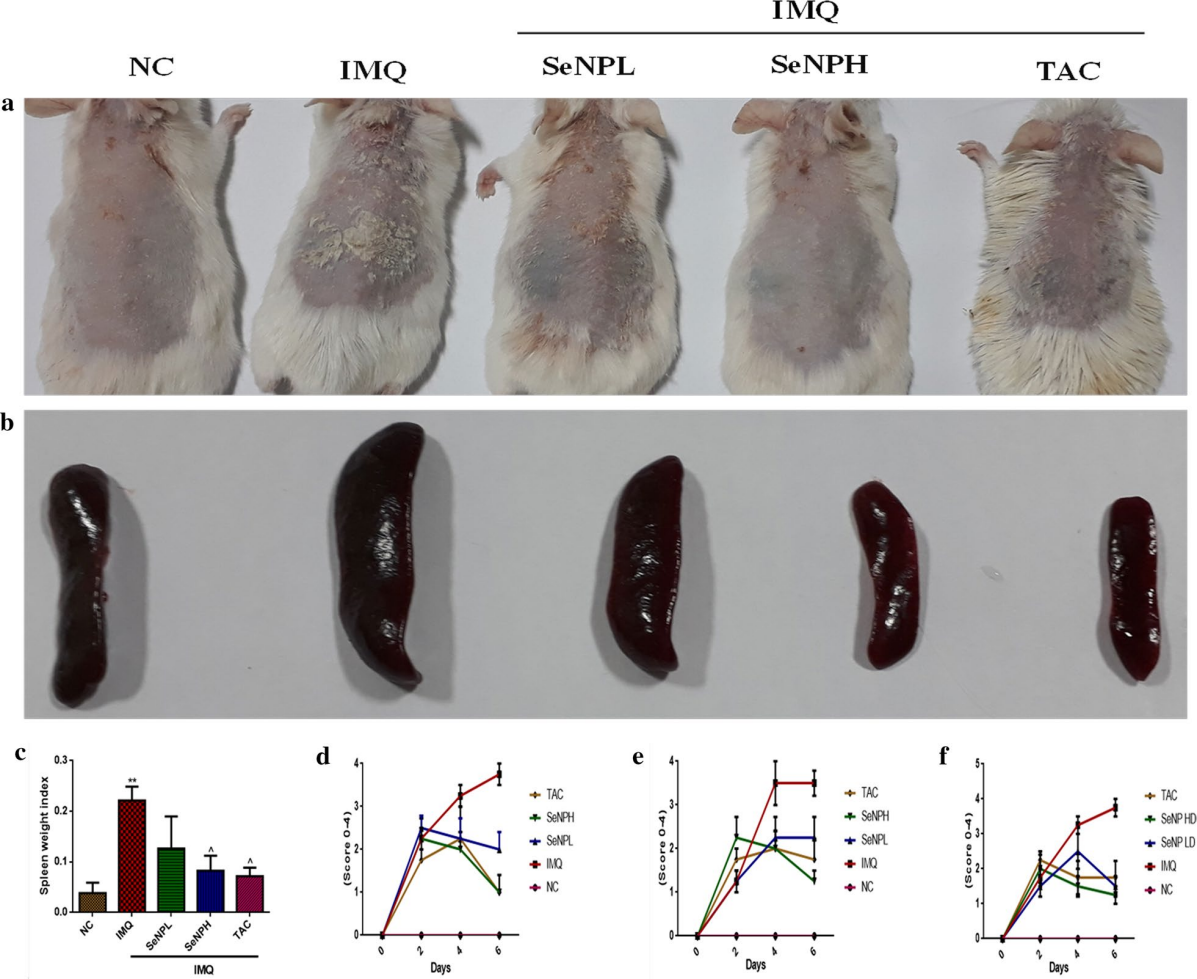
SeNPs inhibit imiquimod (IMQ) induced epidermal hyperproliferation and splenomegaly. **a** Images of IMQ-induced morphological changes in back skin of BALB/c mice with and without SeNPs treatment. **b**, **c** The effect of SeNPs treatment on IMQ induced splenomegaly was recorded by spleen weight by body weight. PASI scoring of the mice with respective to **d** redness, **e** scaling, and **f** thickness on day 0, 2, 4, and 7 were observed and measured. Data represents Mean ± SD (n = 5). **p < 0.01 is significantly different from the normal control group; ^p < 0.05 is significantly different from the IMQ group

On the day of sacrifice, the dorsal region of the skin from all the animal groups was monitored and images were captured. The dorsal skin of the IMQ applied mice group exhibited the signs of erythema after 1 day; the other psoriatic features such as scaling and thickening of the skin were observed after 2–3 days of IMQ application and a gradual increase in the severity was observed until the end of the study in comparison with normal control mice group. SeNPs gel treatment topically at both doses (3 and 10 mg/kg) evidenced improved epidermal structure and the phenotypic changes are comparable to the TAC treated animals (Fig. 5a). On the other side, there was significant enlargement of spleen was observed following 6 days of IMQ application, which depicts the systemic inflammation. Following the treatment, a significant decrease in the IMQ induced splenomegaly was observed and the ratio of spleen weight to body weight index showed a reduction in spleen weights (Fig. 5b, c). As mentioned earlier, the signs of psoriasis (epidermal thickness, erythema, and scaling) on the dorsal skin were evaluated on days 0, 2, 4 and 6. The PASI scoring was graded from 0–4 depending on the severity of symptoms and improvement with the treatment. From Fig. 5d–f, it is clear that SeNPs treatment significantly ameliorated IMQ induced redness and scaling with the reduction in the thickness. Hence, there was a decrease in the scoring on the day 7 similar to TAC**.**

### SeNPs reverse the IMQ-induced acanthosis and improve dermal architecture

**Fig. 6:**
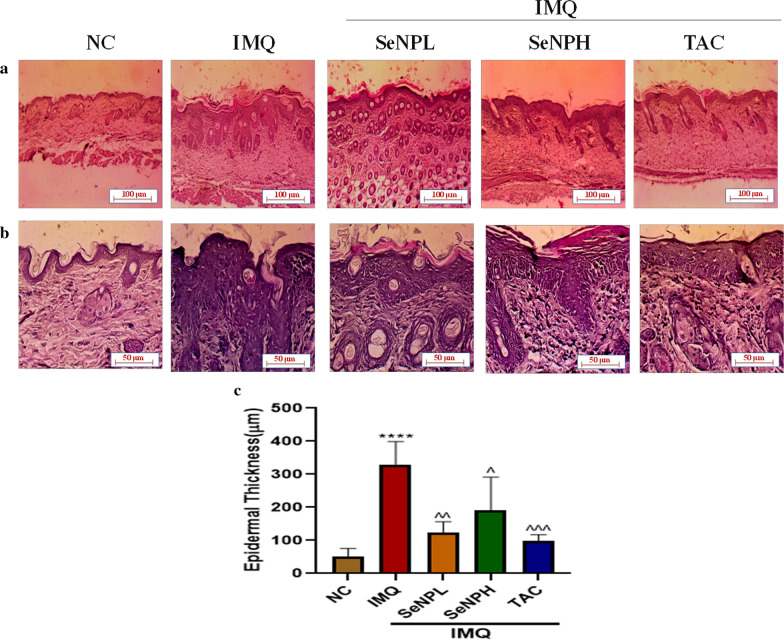
SeNPs inhibit the epidermal thickness. H&E stained skin sections of different treatment groups were observed for evaluation of epidermal thickness in these groups. Topical application of SeNPs significantly inhibited acanthosis in IMQ-induced mice and these images were captured at **a** × 200 and **b** × 400 magnification. (C) Epidermal hyperplasia was measured and represented as bar graph. Data represents Mean ± SD (n = 5). ****p < 0.0001 is significantly different from the normal control group; ^p < 0.05, ^^p < 0.01, and ^^^p < 0.001 is significantly different from the IMQ group

After sacrifice, dorsal skin tissue sections from all the groups were evaluated for histological observations. Consistent with the literature, skin tissue sections from the IMQ group exhibited typical alterations recapitulating psoriasis in mice such as hyperkeratosis, parakeratosis, acanthosis with an elongation of rete ridges compared to control mice. Further, the topical SeNPs gel treatment significantly reduced the epidermal hyperproliferation and improved the epidermal structure to the nearly normal thickness (Fig. 6c) and morphology as compared with the IMQ group and the images were captured at ×200 (Fig. 6a) and ×400 magnification (Fig. 6b).

### SeNPs attenuate the inflammatory cytokines and inhibit inflammatory signaling cascade

**Fig. 7:**
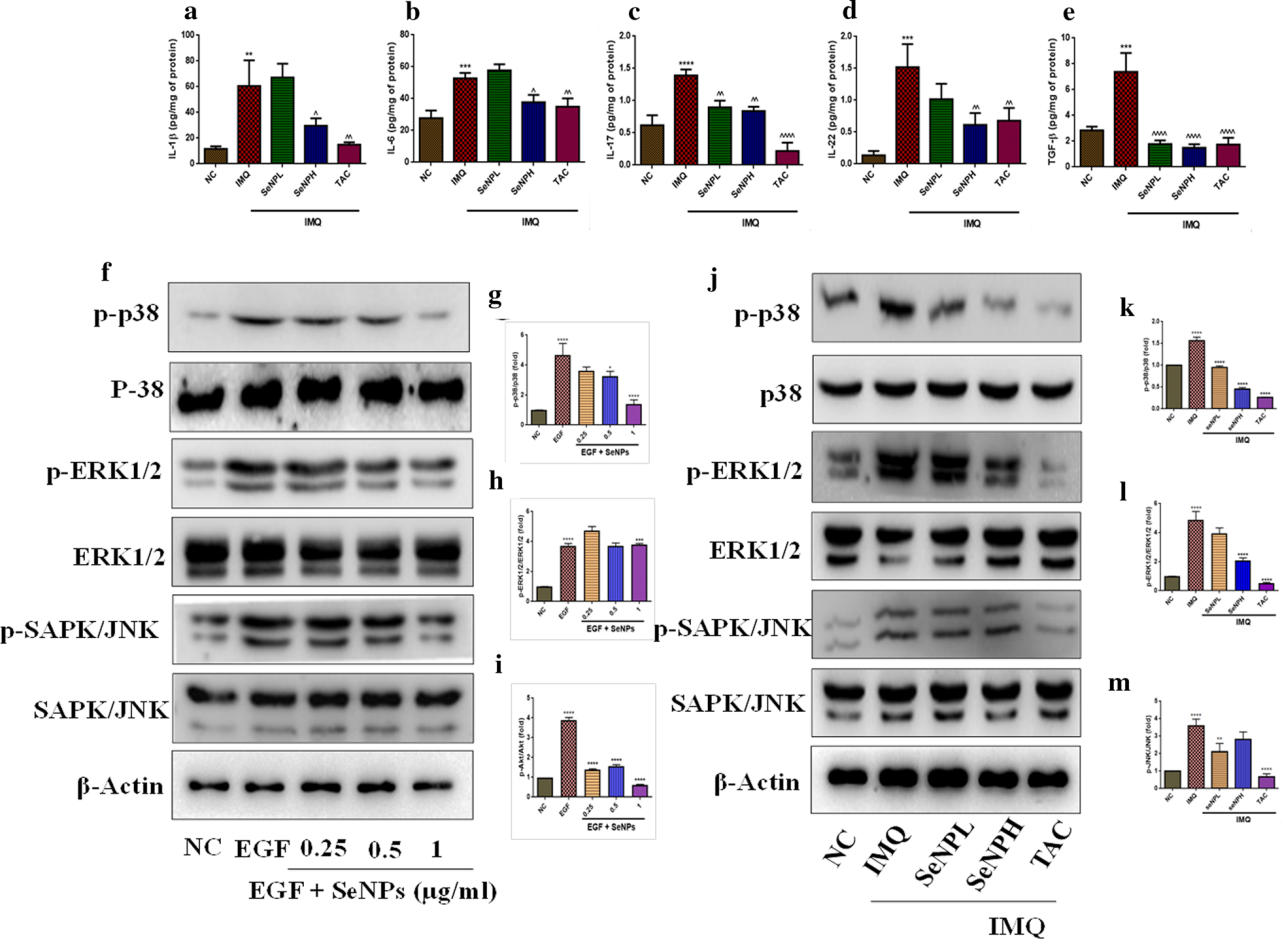
SeNPS inhibit the pro-inflammatory cytokines by suppressing MAPK signaling. The levels of crucial T-cell mediated inflammatory cytokine levels were measured by ELISA. SeNPs treatment ameliorated the levels of these cytokines **a** IL-1β **b** IL-6 **c** IL-17 **d** IL-22 **e** TGF-β, induced by IMQ application and the efficacy was compared with the standard drug TAC. The phosphorylation of p38, p44/42, and JNK was determined in both (**f–i**) HaCaT cells as well as (**j–m**) BALB/C mice. Data represents Mean ± SD (in vitro n = 3; in vivo n = 5). **p < 0.01, ***p < 0.001, and ****p < 0.0001 are significantly different from the normal control group; ^p < 0.05, ^^p < 0.01, ^^^p < 0.001, and ^^^^p < 0.0001 are significantly different from the EGF and IMQ groups

The proinflammatory cytokines play a vital role in psoriasis pathogenesis [49]. Correlating with the existing literature, we found a significant increase in the levels of IL-1β, IL-6, IL-17A, IL-22, and TGF-β cytokines in IMQ skin tissues. Our results demonstrate that these perturbed cytokines levels were significantly attenuated with SeNPs treatment (Fig. 7a–e). MAPKs are involved in signal transduction, proliferation, cell survival and immune-mediated inflammatory responses [50]. It has been demonstrated from the previous reports that there was increased activation of p-44/42, p-SAPK/JNK and p-p38 MAPKs in psoriatic skins. In this study, we investigated possible signaling pathways modulated by SeNPs. Consistent with the previous observations, we found a significant increase in the MAPKs (p-p38, p-SAPK/JNK, and p-p44/42) phosphorylation with EGF stimulation in HaCaT cells (Fig. 7f–i) as well as in the skin tissue sections from IMQ group (Fig. 7j–m). Following the treatment with SeNPs for 24 h followed by EGF stimulation in vitro and SeNPs topical administration in BALB/c mice resulted in decreased activation of MAPKs such as p44/42, p38, and SAPK/JNK phosphorylation in a dose-dependent manner and a decrease in the phosphorylation of these MAPKs were observed.

### SeNPs abrogate hyperproliferation in HaCaT cells and skin tissues

**Fig. 8:**
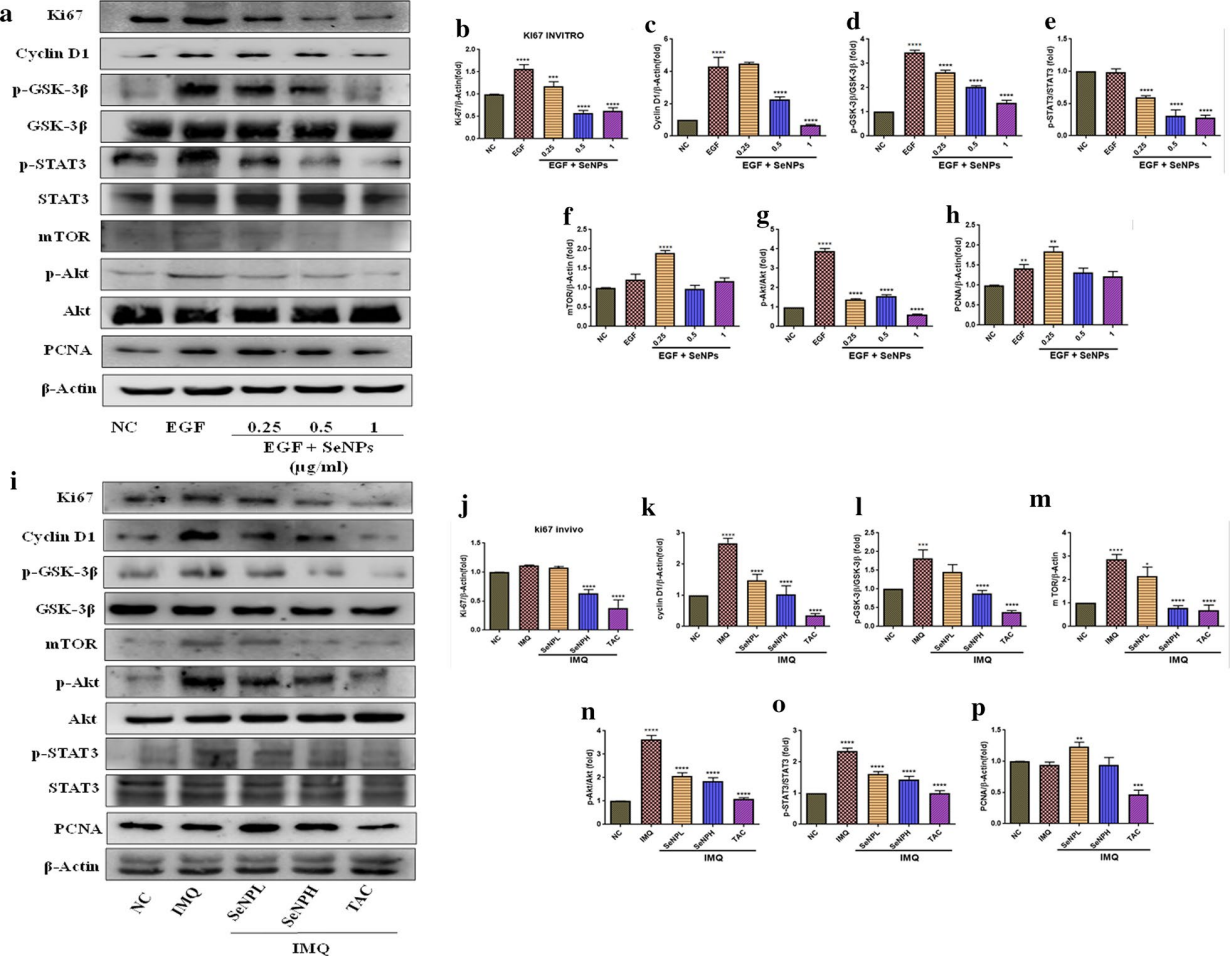
SeNPs inhibit the keratinocyte proliferation and epidermal hyperplasia. **a–h** Cells and **i–p** Mice were treated with SeNPs with various concentrations and protein was extracted to evaluate the protein expression. Ki67, Cyclin D1, PCNA, and mTOR proteins and phosphorylation of Akt, STAT3, and GSK3β was determined by Immunoblotting. Data represents Mean ± SD (in vitro n = 3; in vivo n = 5). *p < 0.05, ***p < 0.001, and ****p < 0.0001 are significantly different from the normal control group; ^^p < 0.01, ^^^p < 0.001, and ^^^^p < 0.0001 are significantly different from the EGF and IMQ groups

**Fig. 9:**
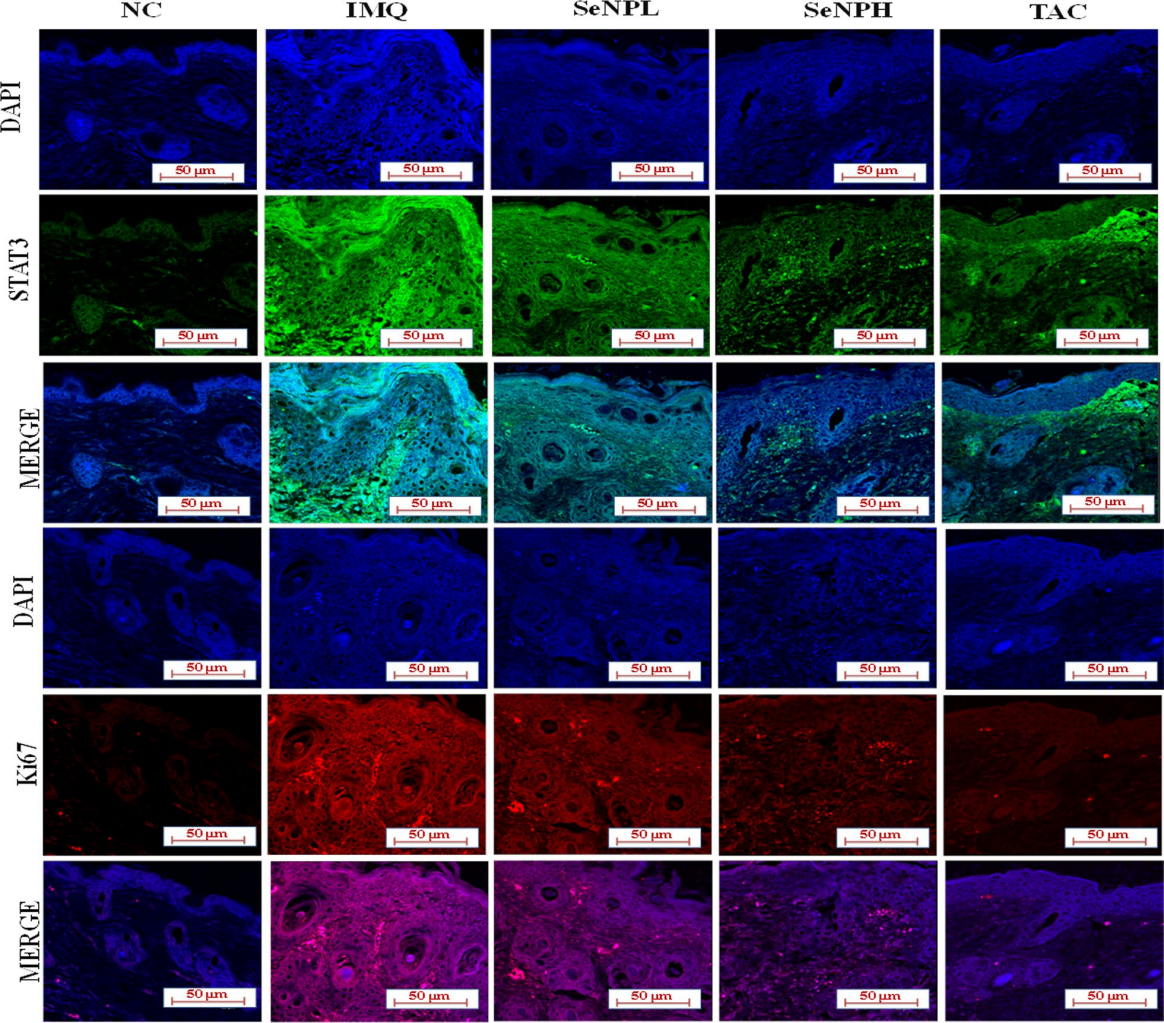
SeNPs downregulate epidermal hyperplasia by suppressing STAT3 and Ki67 expression. Immunofluorescence analysis was performed in skin tissue sections to determine the expression of **a**, **b** STAT3 and **c**, **d** Ki67 using confocal microscopy. The images were captured at × 400 magnification. Data represents Mean ± SD (n = 3). ****p < 0.0001 are significantly different from the normal control group; ^^^^p < 0.0001 are significantly different from the IMQ group

A key phenotypic endpoint in psoriasis is epidermal hyperplasia and the uncontrolled epidermal growth is strongly attributed by cytokines and growth factors which include IL-17, IL-22, EGF, and IGF-1, all together accelerates keratinocyte transit time and mitotic index up to tenfold compared to normal skin [12, 51]. To test the molecular mechanism, HaCaT cells were stimulated with EGF at 50 ng/ml concentration to promote maximal growth and survival, followed by SeNPs treatment (0.25, 0.5, and 1 μg/ml) and then evaluated the protein expression by using western blotting. We observed a marked increase in the expression of proliferative markers expressions such as Ki67, Cyclin D1, and PCNA in EGF stimulated cells (Fig. 8a–h) and IMQ applied skin samples (Fig. 8i–p). A marked downregulation in the expression of these proliferative markers was found with SeNPs treatment. The Akt/mTOR activation and STAT3 signaling are known to promote acanthosis, which is implicated in the immunopathogenesis of psoriasis. In this context, we next investigated the effect of SeNPs on this pathway. We observed a significant increase in the phosphorylation of STAT3 and GSK-3β, while Akt phosphorylation was observed at the Ser473 site. Furthermore, upregulation in the mTOR was observed in EGF and IMQ induction in keratinocytes and mouse skin tissues. The reduced epidermal thickness was further confirmed by immunofluorescence assay was performed in skin tissues from all experimental groups with PCNA and Ki67 antibodies. We found a significant PCNA and Ki-67 immunostaining in the IMQ group, while the expression was found to be reduced in SeNPs treatment at both the doses, indicating the marked reduction in epidermal hyperproliferation and this phenomenon reflects the decreased mitotic index (Fig. 9).

## Discussion

Psoriasis is a chronic autoimmune disease characterized by thickened, scaly plaques as a result of aberrant keratinocyte hyperproliferation, which is accompanied by abnormal differentiation [52]. A vicious cycle between innate immune cells, T cells, and keratinocytes and a complex interplay among them are involved in psoriasis pathogenesis [53, 54]. Among these cells, innate immune cells initiate psoriasis through secretion of IL-23, which has a central role on the type Th17-cell axis [55]. At each step of the psoriasis molecular pathway, different inflammatory cytokines such as IL-6, IL-17, IL-22, and IL-23 are triggered, on the other side, growth factors such as EGF, VEGF, KGF, and IGF-1, which underscores the central role which predominantly drives epidermal hyperplasia [12, 56, 57].

Over the last decade, the treatment options and understanding of the mechanism of psoriasis have witnessed a paradigm shift. However, despite the availability of wide treatment options, the adverse effects associated with long term usage remain a huge problem, which may have a negative impact on patient’s quality of life [58, 59]. Hence, the novel therapeutic options warrant safe and therapeutically effective molecules.

Selenium (Se) is an essential trace element, which is a cofactor for thioredoxin reductase and glutathione peroxidase in animals [23]. The human genome contains 25 selenoprotein genes, which play a crucial role in immunomodulation, sperm motility and also prevent the risk of miscarriage [22, 28]. These selenoproteins, when prepared as nanoparticles, have been used successfully in abrogating several inflammatory disorders [28]. SeNPs exhibited potent anti-cancer properties in treating colorectal, lung and prostate cancers and the main mechanism behind the anti-cancer properties is mediated by induction of apoptosis and cell cycle arrest [60, 61]. A study by Sun D et.al, demonstrated that SeNPs when conjugated with luminescent Ru (II)-thiol selectively inhibited angiogenesis and tumor growth in hepatic carcinoma with minimal host toxicity [62]. Oral administration of SeNPs were found to induce the chemoprotective efficiency against cyclophosphamide induced genotoxicity and hepatotoxicity by the reduction in chromosomal aberrations in bone marrow, and DNA damage in lymphocytes and bone marrow and by balancing the redox state [63]. These fascinating properties of SeNPs prompted us to investigate the effect of SeNPs on immune-mediated benign epidermal hyperproliferation in psoriasis. SeNPs were prepared by the chemical reduction method and the prepared nanoparticles were spherical in morphology and exhibited negative charge. The recent study by Amani et.al, found that c Se nanoparticles has therapeutic effects on ischemic stroke through the protection of axons in hippocampus region, as well as myelination of hippocampal area along with the resolution of brain edema after cerebral ischemic stroke and with minimal side effects, through the regulation of wnt/β-catenin signaling, mTOR, Tsc1/Tsc2 complex, FoxO1 hippo, Ubiquitin–proteasome system (ERK5) which are playing a prominent role in inflammation [64].

The abnormal hyperproliferation of psoriatic keratinocytes resists apoptosis [65–67]. Generally, apoptosis exhibits minimal inflammation and tissue damage; however apoptotic cell death based therapeutics has been exploited as one of the best antipsoriatic therapy. Previous reports demonstrated that apoptosis induction is the key mechanism behind the successful regression of psoriatic hyperplasia with conventional PUVA and methotrexate therapy [33, 34]. To test a similar possibility initially, we have evaluated the effect of SeNPs on cell viability. Here, the hyperproliferation state of keratinocytes was mimicked by stimulating the cells with EGF and then cells were treated with SeNPs, where a gradual dose-dependent inhibition in the cell viability was observed. Next, we investigated the effect of SeNPs on apoptosis induction and we found prominent changes in the morphology of the cells with apoptotic features such as cytoplasmic shrinkage, nuclear fragmentation evident from AO and DAPI fluorescent staining as well as phase-contrast imaging.

The increase in ROS generation leads to the simultaneous collapse of ΔΨm and mitochondrial dysfunction. On the other side, intracellular ROS accumulation impedes cell proliferation by arresting cell cycle phases [68]. Previous reports showed that SeNPs can function as both prooxidant as well as antioxidant which is dependent on the dose and disease condition. At non lethal doses, Se typically induces apoptosis along with growth inhibitory properties by acting as prooxidant that induces oxidative stress in various cancers [69–73]. On the other side, in an ischemia reperfusion injury model it was found that Se NPs can effectively act as an antioxidant through the scavenging of different free radicals such as singlet oxygen, superoxide anion as well as nitric oxide [74]. The effect of SeNPs on HaCaT cells redox state with EGF stimulation was quantified by staining the cells with DCFDA, JC-1 and PI stain based cell cycle analysis. Our results showed that SeNPs treatment significantly elevates the levels of ROS in EGF stimulated HaCaT cells which were evaluated by a shift in the peak towards the green signal (right), which corresponds to high DCF molecules produced by the total cellular ROS by oxidation of DCFDA dye. Furthermore, JC-1 stain incorporates into healthy mitochondria forms J aggregates and fluoresces orange-red. When ΔΨm collapses, JC-1 will remain as J-monomers and exhibit green fluorescence. We observed a concentration dependent dissipation of ΔΨm with an increase in the mitochondrial membrane depolarization. The formation of smaller base pair DNA fragments and single-strand cleavage events are the biochemical hallmarks of apoptosis [75]. From the cell cycle analysis data, a sharp increase in the discrete histogram peak representing the sub-G1 population was observed in SeNPs treated cells, compared to the control cell population. Next, annexin V Alexa flour 488 /PI assay was performed to detect early and late apoptosis in support of the previous results. Where, it was found that SeNPs treatment induced significant early apoptosis with an increase in concentration, while moderate late apoptosis was observed with negligible necrosis. Collectively, the present study suggests that SeNPs exerts its mechanism by a strong induction of apoptosis in EGF induced epidermal keratinocytes.

To investigate the effect of SeNPs on psoriasis in vivo, IMQ was used to induce psoriasis which is a widely accepted animal model, acts by binding to Toll-like receptors (TLR7 and TLR8), which involved in activation of innate immunity and stimulates adaptive immunity and recapitulates typical histopathological features in mice such as parakeratosis, acanthosis, scaling, erythema, skin thickening, and inflammatory cell infiltration which closely resembles human psoriasis [76]. Consistent with the previous literature, we have observed similar psoriatic features in mice upon the IMQ application. SeNPs were treated topically which were incorporated in a gel at 3 and 10 mg/kg doses respectively. Interestingly, our in vivo results evidenced that SeNPs daily topical application at both doses resulted in a reduction in plaque formation, erythema, and scaling. There was an increase in the skin thickness observed with IMQ application in both the dorsal region and left ear measured by vernier calipers, while in treatment groups a decrease in both skin and ear thickness was observed and the results are comparable with the standard TAC. Based on these observations PASI scoring (erythema, thickening, and scaling) was given every alternate day. In IMQ applied animal groups a marked pathological change was observed in the epidermis and/or dermis such as an increase in epidermal keratinocytes protruded into the dermis as rete ridges with prominent parakeratosis along with the recruitment of leukocyte subsets into the skin. Microscopic evaluation SeNPs treated skin tissue sections underlined the superior ability in alleviating these pathological consequences. The systemic immune status is envisaged by the spleen, which is the largest immune organ, the state of inflammation in psoriasis enlarges the spleen due to a huge increase in the number of cells such as Th17/Th22 T cells [77]. Correlating with the previous studies, we have found a significant enlargement of spleen up to 3-folds compared to the normal control group. Topical SeNPs treatment significantly reduced the splenomegaly, which is indicative of alleviation in the inflammatory response.

The activation and upregulation of cytokines and chemokines produces a “feed-forward” inflammatory response in keratinocytes. IL-1β together with IL-23 are crucial in inducing Th17 and Th22 cell differentiation which further produces IL-17 and IL-22 cytokines. While IL-6 cytokine primes Th17 in mediating hyper-proliferation in conjunction with IL-23 cytokine [78]. Previous reports suggest that STAT3 plays a crucial role in Th17 and Treg cell differentiation and deregulation of STAT3 leads to the psoriasis pathogenesis through mediation of IL-17/IL-22/ IL-23 axis signaling; also the cytokines such as IL-6 and IL-21 are critical for Th17 cells maintenance that functions mainly through STAT3, also an impaired IL-17 production was observed in the absence of STAT3 [79, 80]. On another hand, a study by Han et al., reported the expression of wild-type TGFβ1 in the epidermis developed severe skin inflammation [81]. In support of this literature, our results evidenced a significant elevation in the aforementioned cytokines in the IMQ group. Moreover, SeNPs treatment significantly suppressed IL-1β, IL-6, IL-17, IL-22, and TGF-β cytokine levels. The aberrant activation of MAPK kinases which includes p38, ERK1/2, and JNK are involved in the pathogenesis of psoriasis [82]. As described above, following the previous studies increased phosphorylation levels of the p44/42, SAPK/JNK, and p38 proteins were observed following EGF treatment, as well as in skin tissues and these effects on phosphorylation were alleviated by SeNPs. Previous studies have shown that PI3K/Akt/mTOR and STAT3 are the main orchestrators of the immune response and keratinocyte hyperproliferation that sustains the chronic epidermal hyperplasia [83, 84]. In agreement with previous studies, both EGF stimulation and IMQ application increased these signaling cascades. On the other side, upregulation of Ki-67, PCNA and Cyclin D1 are the important hyperproliferative diagnostic hallmarks which differentiate psoriatic skin from normal skin. Molecularly, SeNPs treatment reduced the expression of cell growth promoters and proliferation markers such as Ki-67, PCNA, cyclin D1, and mTOR. A substantial reduction in Akt at ser 473, GSK-3β, and STAT3 at Tyr 708 site was observed both in vitro and in vivo samples*.* Furthermore, we found that STAT3 and Ki67 activation in terms of localization and expression level in the thicker plaques of the epidermis of the IMQ group when compared to the normal epidermis with strong positive staining evaluated by immunofluorescence. Whereas SeNPs treated group showed a reduction in the immunopositivity and these results are in par with the standard TAC group.

**Fig. 10:**
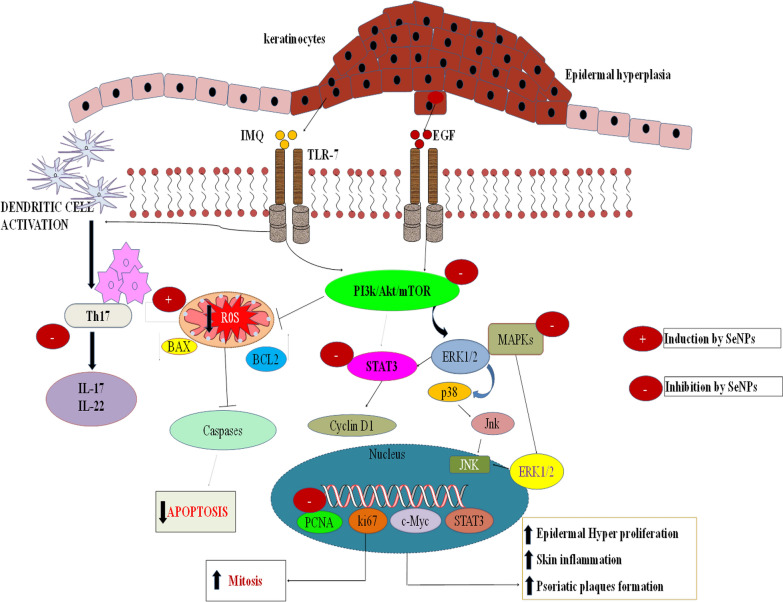
SeNPs ameliorate the psoriatic like skin disease by suppressing keratinocyte hyperproliferation and inflammation. A schematic diagram depicts the mechanism of SeNPs in psoriatic like skin disease. IMQ activates the TLR-7 and Th17, which results in epidermal hyperplasia and keratinocyte hyperproliferation. Whereas, EGF induces the Akt/mTOR/MAPKs signaling and further enhance the cell proliferation and inflammation. IMQ and EGF induced epidermal proliferation and inflammation is prevented by SeNPs by different canonical pathways

In summary, our findings demonstrate that SeNPs primarily target keratinocytes in vitro by inducing apoptosis and thereby reduce keratinocytes hyperproliferation. Whereas, our in vivo findings demonstrate the effectiveness of SeNPs in alleviating IMQ induced acanthosis and further abrogated cytokines levels by targeting various signaling events involved in the psoriasis pathogenesis (Fig. 10).

## Conclusion

Although many therapies are available as treatment options in psoriasis armamentarium, still there is an unmet need for new drugs with high efficacy and limited side effects. To the best of our knowledge, we report for the first time that SeNPs inhibit epidermal hyperplasia with potent anti-proliferative and anti-inflammatory activities. Our findings strongly suggest that SeNPs may be a potential therapeutic alternative in the treatment of psoriasis; the biological inertness is one of the advantages of SeNPs which may possess greater relevance in psoriasis with less adverse effects.

## Data Availability

The datasets used and/or analyzed during the current study are available from the corresponding author on reasonable request.
